# ^3^H-Deprenyl and ^3^H-PIB autoradiography show different laminar distributions of astroglia and fibrillar β-amyloid in Alzheimer brain

**DOI:** 10.1186/1742-2094-10-90

**Published:** 2013-07-23

**Authors:** Amelia Marutle, Per-Göran Gillberg, Assar Bergfors, Wenfeng Yu, Ruiqing Ni, Inger Nennesmo, Larysa Voytenko, Agneta Nordberg

**Affiliations:** 1 Alzheimer Neurobiology Center, Department of Neurobiology, Care Sciences and Society, Karolinska Institutet, Novum Floor-5, Stockholm S-14186, Sweden; 2 Department of Geriatric Medicine, Karolinska University Hospital Huddinge, Stockholm, Sweden; 3 Department of Pathology, Karolinska University Hospital Huddinge, Stockholm, Sweden; 4 Douglas Mental Health University Institute, Department of Psychiatry, McGill University, Montreal, Quebec, Canada

**Keywords:** Alzheimer’s disease, Postmortem brain, Laminar pathology, Astrogliosis, Microgliosis, Fibrillar amyloid, Nicotinic acetylcholine receptors, PIB, Quantitative autoradiography

## Abstract

**Background:**

The pathological features in Alzheimer’s disease (AD) brain include the accumulation and deposition of β-amyloid (Aβ), activation of astrocytes and microglia and disruption of cholinergic neurotransmission. Since the topographical characteristics of these different pathological processes in AD brain and how these relate to each other is not clear, this motivated further exploration using binding studies in postmortem brain with molecular imaging tracers. This information could aid the development of specific biomarkers to accurately chart disease progression.

**Results:**

*In vitro* binding assays demonstrated increased [^3^H]-PIB (fibrillar Aβ) and [^3^H]-PK11195 (activated microglia) binding in the frontal cortex (FC) and hippocampus (HIP), as well as increased binding of [^3^H]-l-deprenyl (activated astrocytes) in the HIP, but a decreased [^3^H]-nicotine (α4β2 nicotinic acetylcholine receptor (nAChR)) binding in the FC of AD cases compared to age-matched controls. Quantitative autoradiography binding studies were also performed to investigate the regional laminar distributions of [^3^H]-l-deprenyl, [^3^H]-PIB as well as [^125^I]-α-bungarotoxin (α7 nAChRs) and [^3^H]-nicotine in hemisphere brain of a typical AD case. A clear lamination pattern was observed with high [^3^H]-PIB binding in all layers and [^3^H]-deprenyl in superficial layers of the FC. In contrast, [^3^H]-PIB showed low binding to fibrillar Aβ, but [^3^H]-deprenyl high binding to activated astrocytes throughout the HIP. The [^3^H]-PIB binding was also low and the [^3^H]-deprenyl binding high in all layers of the medial temporal gyrus and insular cortex in comparison to the frontal cortex. Low [^3^H]-nicotine binding was observed in all layers of the frontal cortex in comparison to layers in the medial temporal gyrus, insular cortex and hippocampus. Immunohistochemical detection in the AD case revealed abundant glial fibrillary acidic protein positive (GFAP^+^) reactive astrocytes and α7 nAChR expressing GFAP^+^ astrocytes both in the vicinity and surrounding Aβ neuritic plaques in the FC and HIP. Although fewer Aβ plaques were observed in the HIP, some hippocampal GFAP^+^ astrocytes contained Aβ-positive (6 F/3D) granules within their somata.

**Conclusions:**

Astrocytosis shows a distinct regional pattern in AD brain compared to fibrillar Aβ, suggesting that different types of astrocytes may be associated with the pathophysiological processes in AD.

## Introduction

The gradual accumulation of β-amyloid (Aβ) peptides in the brain, varying in size and state of aggregation, is suggested to play a central role in Alzheimer’s disease (AD), triggering a cascade of neurodegenerative changes in the brain. These include neurofibrillary tangle formation, the activation and exacerbation of inflammatory processes, impairment of neurotransmitter signaling, and the perturbation of synaptic functions resulting in the death of neurons in brain areas associated with learning and memory [1,2].

The rapid development of molecular imaging in the past decade has provided valuable new tools for the understanding of complex disease mechanisms in AD. Positron emission tomography (PET) imaging of the brain using amyloid tracers has provided evidence that the accumulation of fibrillar Aβ in the brain occurs early on in the disease course, preceding progressive changes in metabolic activity and structure, which occur closer to the manifestation of clinical symptoms in AD [3-9].

AD-associated inflammation has been widely described by pathological examination of brain tissue from AD patients demonstrating abundant activated microglia in Aβ plaques and increased numbers of reactive astrocytes surrounding Aβ plaques [10-15]. However, it is not clear whether the inflammatory response detected in postmortem brain was a cause or a consequence of disease progression. It is suggested that the inflammatory processes in AD may have contrasting roles where, for instance, activated glia not only eliminate Aβ plaques via phagocytosis but may also initiate a proinflammatory cascade that results in the release of potentially neurotoxic substances such as cytokines, complement components, various free radicals, and nitric oxide, all of which may contribute to further neuronal dysfunction and cell death [16]. Findings from the most recent multitracer PET studies in patients with mild cognitive impairment (MCI) and mild AD indicate that astrocytosis is similar to Aβ accumulation, an early phenomenon in AD, but follows a different spatial and temporal pattern than fibrillar Aβ deposition and impaired synaptic activity as measured by glucose metabolism [17].

Although *in vivo* imaging methods provide valuable quantitative information with regards to disease progression and understanding the complex pathology in AD neurodegeneration, it is also important to study in autopsy brain how the different pathological processes are related.

In the present study, we investigated the relationship between regional neuroinflammatory processes, fibrillar Aβ deposition, and disturbances in cholinergic neurotransmission in AD brain. Binding studies were carried out in postmortem brains from a group of age-matched AD and non-demented control cases with the radioligands [^3^H]-l-deprenyl (activated astrocytes), [^3^H]-PIB (fibrillar Aβ), [^3^H]-PK11195 (microglia) as well as [^125^I]-α-bungarotoxin (α7 nicotinic receptors, nAChRs) and [^3^H]-nicotine (α4β2 nAChRs). We also applied an *in vitro* imaging multitracer concept in order to characterize and compare the laminar distributions of activated astrocytes, fibrillar Aβ, as well as α7 and α4β2 nAChRs in hemisphere brain sections of an AD patient who was clinically followed at regular intervals until death.

## Methods

### Subjects

Postmortem brain tissues from the superior frontal gyrus and the hippocampus from 11 AD cases (age 75.2 ± 2.7 years; postmortem delay 15.9 ± 3.2 h; Braak stages 5 to 6), and 13 age-matched controls (age 73.9 ± 3.0 years; postmortem delay 18.5 ± 2.5 h; Braak stages 1 to 2) were obtained from the Brain Bank at Karolinska Institutet and the Netherlands Brain Bank. Each AD case had a clinical diagnosis of AD confirmed by pathological examination according to criteria from the National Institute of Neurological and Communicative Disorders and Stroke and the Alzheimer’s disease and Related Disorders Association (NINCDS-ADRDA) and Consortium to Establish a Registry for Alzheimer’s disease (CERAD) workgroups. The control cases had no history of psychiatric or neurological disorders or neuropathology indicating dementia. The main cause of death among the AD cases was bronchopneumonia and for controls, myocardial infarction. Permission to use autopsy brain material in experimental procedures was granted by the Regional Human Ethics committee in Stockholm and the Swedish Ministry of Health. All material and data collected by the Netherlands Brain Bank were obtained on the basis of written informed consent.

### Binding assays

Brain samples from the frontal cortex and hippocampus of AD and control cases were homogenized in cold phosphate-buffered saline (PBS) pH 7.0 containing protease inhibitors. Triplicate samples of prepared crude homogenates were incubated with 1 nM [^3^H]-PIB (SA 68 Ci/mmol), 10 nM [^3^H]-l-deprenyl (SA 80 Ci/mmol), 5.0 nM [^3^H]-PK11195 (specific activity 83.4 Ci/mmol, American Radiolabeled Chemicals, St. Louis, MO, USA), as previously described [6,18]. For the [^3^H]-nicotine (5 nM; SA 75 Ci/mmol) and [^125^I]-α-bungarotoxin (2 nM; SA 108.8 Ci/mmol) binding assays to α4β2 and α7 nAChRs, respectively, membrane fractions were prepared by homogenization in 0.32 M sucrose, centrifugations and resuspension in binding buffer prior to incubations. Specific binding was expressed in fmol/mg tissue or fmol/mg protein.

### Cryosectioning

From the set of AD cases described above, a 61-year-old AD case (17 h postmortem delay; Braak stage 6) was selected for large-section cryomicrotomy.

Guided by an atlas of the human brain [19], 5-mm thick coronal planes situated at a distance of 30 to 40 mm from the most anterior part of the brain were chosen for this study. From these planes, 80-μm thick sections from the left hemisphere were cut at −20°C and thaw-mounted on gelatin-coated 200 × 150 mm^2^ glass plates, as described previously [20].

### Whole hemisphere autoradiography

Brain sections were thawed at room temperature (RT) prior to incubations. Autoradiography binding studies were performed by preincubating sections for 15 minutes in PBS buffer (pH 7.4) followed by incubation with 5 ml of respective ligand; 1 nM [^3^H]-PIB (specific activity, SA 68 Ci/mmol, custom synthesis; GE Healthcare, Freiburg, Germany) for 45 minutes [21]; 10 nM [^3^H]-l-deprenyl (specific activity 80 Ci/mmol, Larodan Fine Chemicals AB, Malmö, Sweden) for 1 h [22]; 5.0 nM [^3^H]-nicotine (specific activity 75 Ci/mmol, NEN Life Science Products, Dreiech, Germany) for 40 minutes [23]; and 2 nM [^125^I] α-bungarotoxin (specific activity 108.8 Ci/mmol, Perkin Elmer, Waltham, MA, USA) for 30 minutes [24]. To determine non-specific binding for each radioligand, adjacent sections were incubated with 1 μM of unlabeled PIB, 1 μM unlabeled deprenyl, 100 μM (−) nicotine or 1 μM unlabeled α-bungarotoxin (Tocris Bioscience, Bristol, UK), respectively. The binding reactions were terminated by rinsing sections 3 × 5 minutes in cold buffer and a brief rinse in deionized distilled water. Sections were air dried for at least 48 h and placed together with calibrated tritium standards in autoradiography cassettes and exposed to Fujifilm BAS-2500 imaging plates (Science Imaging Scandinavia AB, Nacka, Sweden) or to tritium-sensitive film (Amersham Hyper film MP; GE Healthcare) for the appropriate length of time. For [^125^I]-α-bungarotoxin autoradiography, different concentrations of [^125^I]-α-bungarotoxin (0.5 to 5 nM), serving as a standard, were applied onto a filter paper and exposed together with the sections to Kodak Biomax MR film (Sigma, St Louis, MO, USA) for 14 days.

### Quantitative analysis of autoradiographic images

All laminar identification examinations were carried out with guidance of a classical atlas Brodman [25] and Nissl stained sections, and further assisted by autoradiograms of [^3^H]-l-deprenyl binding, since [^3^H]-l-deprenyl has earlier been shown to have a clear demarcation between different laminae in human brain cortex [22,26].

For quantification, the optical densities were measured using computer-assisted image analysis that digitized the light intensity into 256 levels (Imteck Vision, Uppsala, Sweden). A calibration curve was obtained from a set of films with known optical densities (OD) (KODAK Wratten gelatin filters). The unit area (pixel) of measurement was about 180 μm^2^. The digitized picture was displayed on a color monitor and regions for calculation of mean OD were selected. For each region, three autoradiograms were analyzed and two readings were taken. The binding density (fmol/mg tissue) corresponding to the autoradiography obtained gray values in the cortical areas was determined from the micro scale standards that contained a known amount of radioactivity per mass of tissue, and divided by the specific radioactivity of each ligand. To measure the laminar distribution of binding densities in the cortex, averaged gray levels of 20 or more consecutive lines (corresponding to a total width of ≥0.65 mm) perpendicular to the entire cortical depth from the pial surface to the white matter were determined as described by [23]. Profiles were created at the position of each examined cortical area where the highest quality of tissue was observed. Accordingly, differences could occur in the width of the same layer within one cortical region. The entire depth of the cortex was standardized to 100% to account for variances in the absolute cortical depth, due to a slight change of angle during tissue sectioning. The non-specific binding, which did not show any laminar pattern, was subtracted.

### Immunohistochemistry

Immunohistochemistry was performed on the single AD case by pretreating sections with formic acid (88%) for 10 minutes or with ethylenediaminetetra-acetic acid, (EDTA) for 20 minutes. For antigen retrieval, sections were heated in a microwave oven (700 W) for 10 minutes in 0.05 M citrate-buffered saline (pH 6.0) followed by a blocking step. The sections were incubated with antibodies specific for Aβ 6 F/3D (1:200 to 1:400; Dako, Glostrup, Denmark) or 4G8 (1:200, Chemicon International, Temecula, CA, USA), Tau AT8 (1:500, Innogenetics, Gent, Belgium), microglia CD68 (1:100, Dako), glial fibrillary acidic protein (GFAP; 1:300 to 1:500, Dako), mouse monoclonal α7 nAChR mAb 306 antibody (1:1,000, Sigma-Aldrich), and biotin-conjugated α-bungarotoxin (Invitrogen, Carlsbad, CA, USA). Following incubation with biotinylated secondary antibodies and washes, the VECTASTAIN Elite ABC (Vector Laboratories, Burlingame, CA, USA), chromogen Vector SG substrate or EnVision™ G|2 System/AP Rabbit/Mouse (Permanent Red visualization (Dako) kits, respectively were used for detection. Controls consisted of brain sections treated with either non-immune serum or omission of primary antibody.

For analysis of histological findings, an image analysis system was utilized that allowed processing of images from a video camera attached to a microscope. The percentage of astrocytes expressing the α7nAChR subunit was assessed in double-stained sections as previously described [27]. For each brain section, six strips (with a total width of 600 μm) that extended from the pial surface to the border with the white matter were chosen from the top, middle, and bottom of the sections for evaluation.

## Results

### Comparison of binding levels for fibrillar amyloid, reactive astrocytes, and activated microglia in relation to α4β2 and α7 nicotinic receptors in AD and control brain

**Figure 1:**
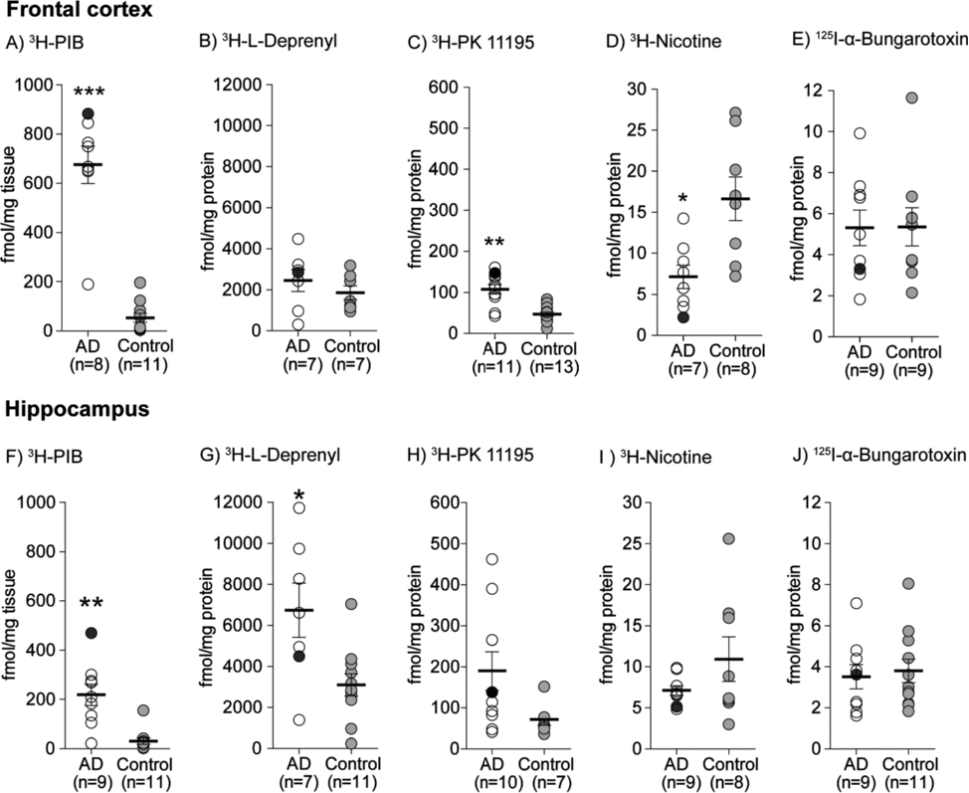
**Comparison of binding levels of (A, F) [**^**3**^**H]-PIB (fibrillar β-amyloid), (B, G) [**^**3**^**H]-l-deprenyl (activated astrocytes), and (C, H) [**^**3**^**H]-PK11195 (activated microglia), as well as (D, I) [**^**3**^**H]-nicotine and (E, J) [**^**125**^**I]-α-bungarotoxin to α4 and α7 nicotinic acetylcholine receptor (nAChR) subtypes, respectively, in the frontal cortex and hippocampus of Alzheimer’s disease (AD) cases (open circles) and age-matched controls (filled circles).** Data obtained from the binding assays are expressed in fmol/mg tissue or protein (means ± SEM), and were analyzed by one-way analysis of variance (ANOVA) followed by Bonnferroni’s or Dunnet’s post hoc tests to compare statistical differences between control and AD cases. *P <0.05; **P <0.01; ***P <0.001.

Significantly higher binding levels of [^3^H]-PIB were measured both in the frontal cortex (*P* <0.0007) and in the hippocampus (*P* <0.002) of AD cases compared to age-matched controls (Figure 1A,F). Similarly, significant increases in the binding of [^3^H]-l-deprenyl in the hippocampus (*P* <0.03) and [^3^H]-PK11195 in the frontal cortex (*P* <0.002) were observed in AD cases (Figure 1C,G). [^3^H]-Nicotine binding was significantly reduced in the frontal cortex (*P* <0.02) of AD cases (Figure 1D), while no significant changes in [^125^I]-α-bungarotoxin binding were observed in either the frontal or hippocampus of AD cases relative to controls (Figure 1E,J).

### Laminar autoradiographical distributions fibrillar amyloid, reactive astrocytes, and nicotinic receptor subtypes in AD brain

**Figure 2:**
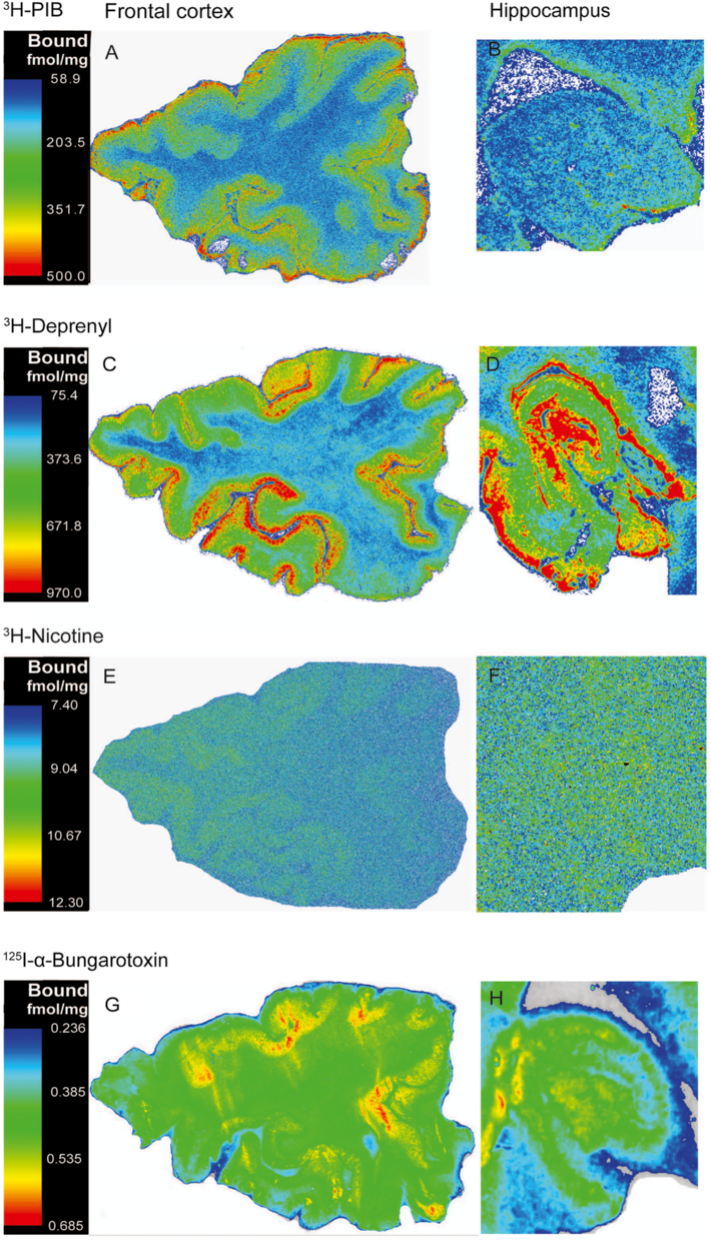
**Representative pseudocolored autoradiographical distributions of (A,B) [**^**3**^**H]-PIB, (C,D) [**^**3**^**H]-l-deprenyl, (E,F) [**^**3**^**H]-nicotine, and (G,H) [**^**125**^**I]-α-bungarotoxin binding in coronal sections obtained from the frontal cortex and hippocampus of a typical Alzheimer’s disease (AD) patient at autopsy.** The density of the binding increases along the sequence of blue, green, yellow, and red. The pseudocolored figures are not standardized to each other, since the series of autoradiograms for each ligand were created individually.

Autoradiography binding in coronal hemisphere brain sections from the AD case showed varied distribution profiles for [^3^H]-PIB, [^3^H]-l-deprenyl, [^3^H]-nicotine, and [^125^I]-α-bungarotoxin as illustrated in the pseudocolored images in Figure 2.

**Figure 3:**
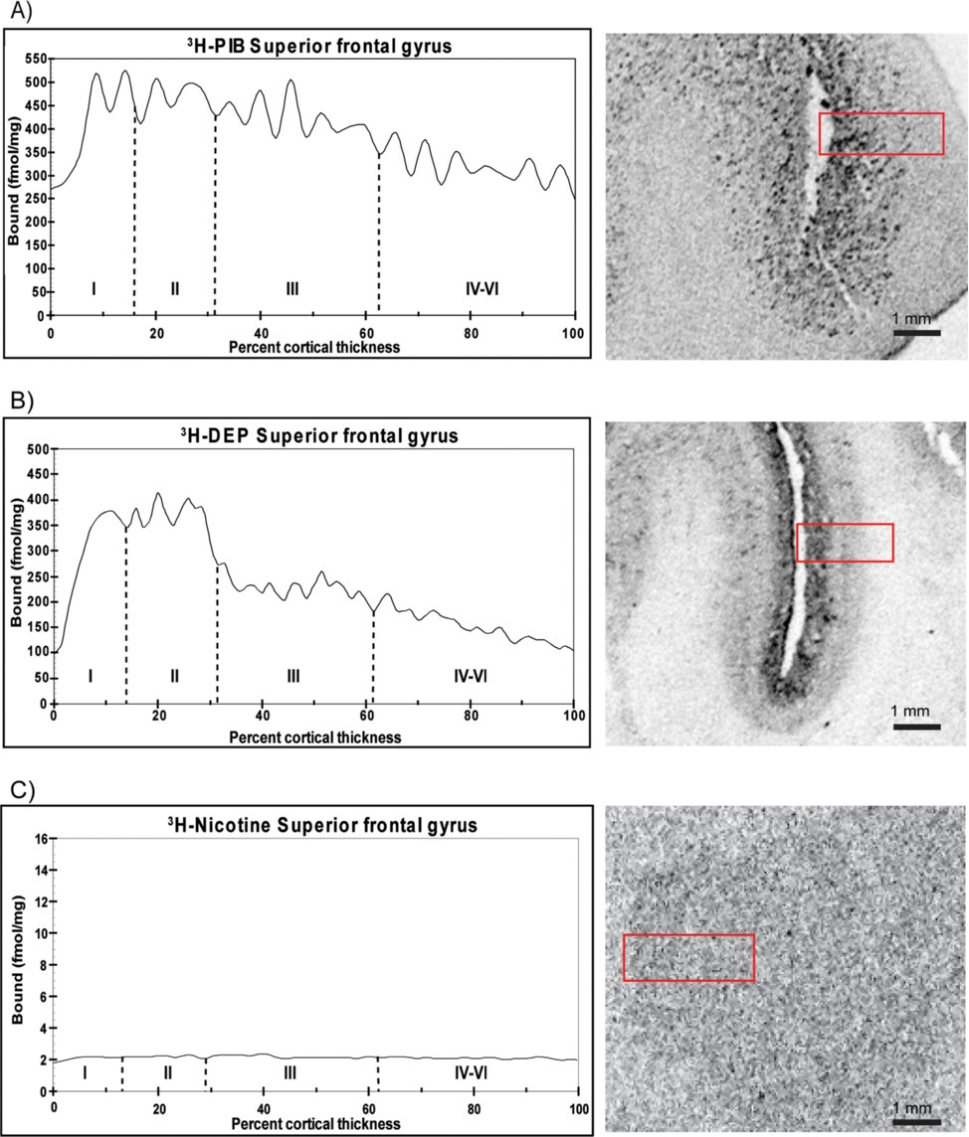
**Laminar distribution of total binding for (A) [**^**3**^**H]-PIB, (B) [**^**3**^**H]-l-deprenyl, and (C) [**^**3**^**H]-nicotine throughout the entire cortical depth of the superior frontal gyrus in an Alzheimer’s disease (AD) case.** The binding profiles are means created from three consecutive sections.

**Figure 4:**
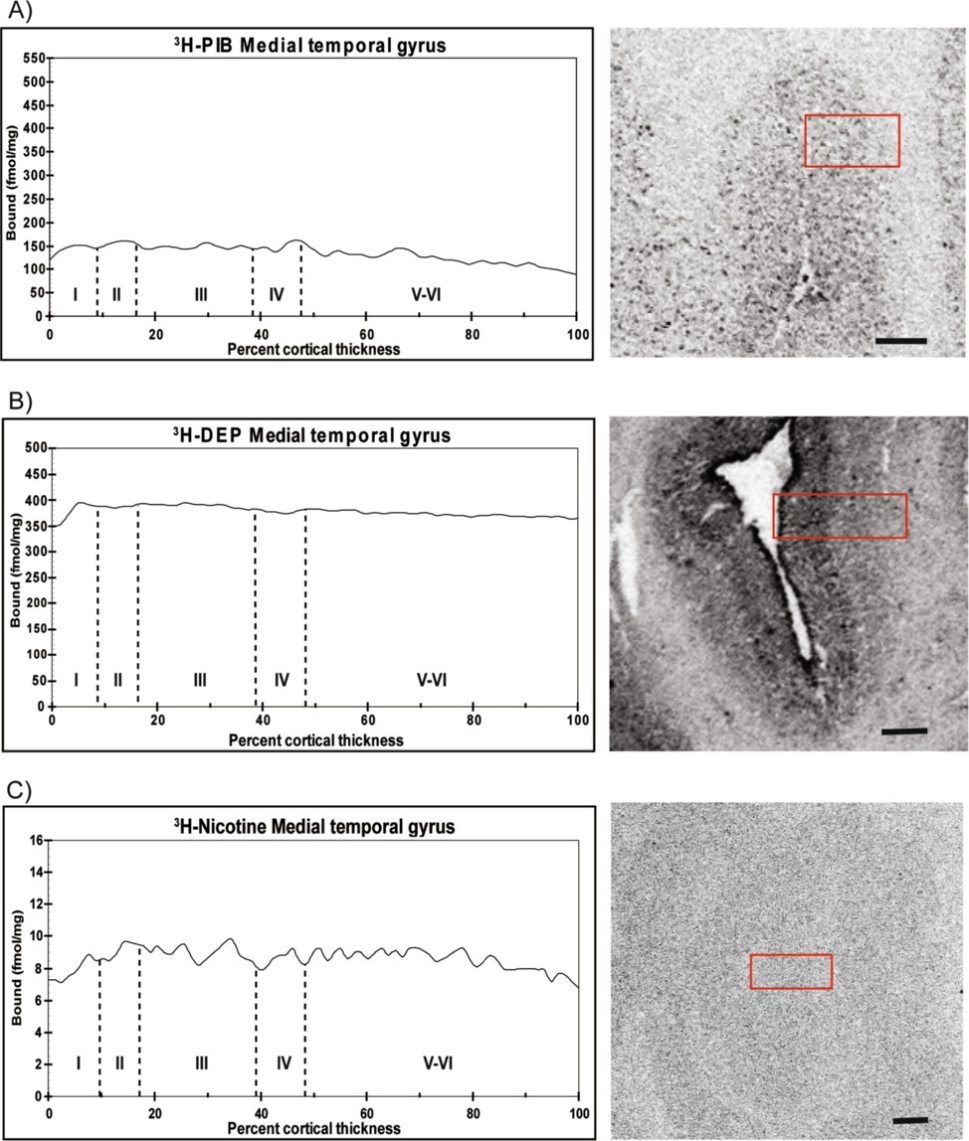
**Laminar distribution of (A) [**^**3**^**H]-PIB, (B) [**^**3**^**H]-l-deprenyl, and (C) [**^**3**^**H]-nicotine total binding throughout the entire cortical depth of the medial temporal gyrus in an Alzheimer’s disease (AD) case.** The binding profiles are means created from three consecutive sections.

**Figure 5:**
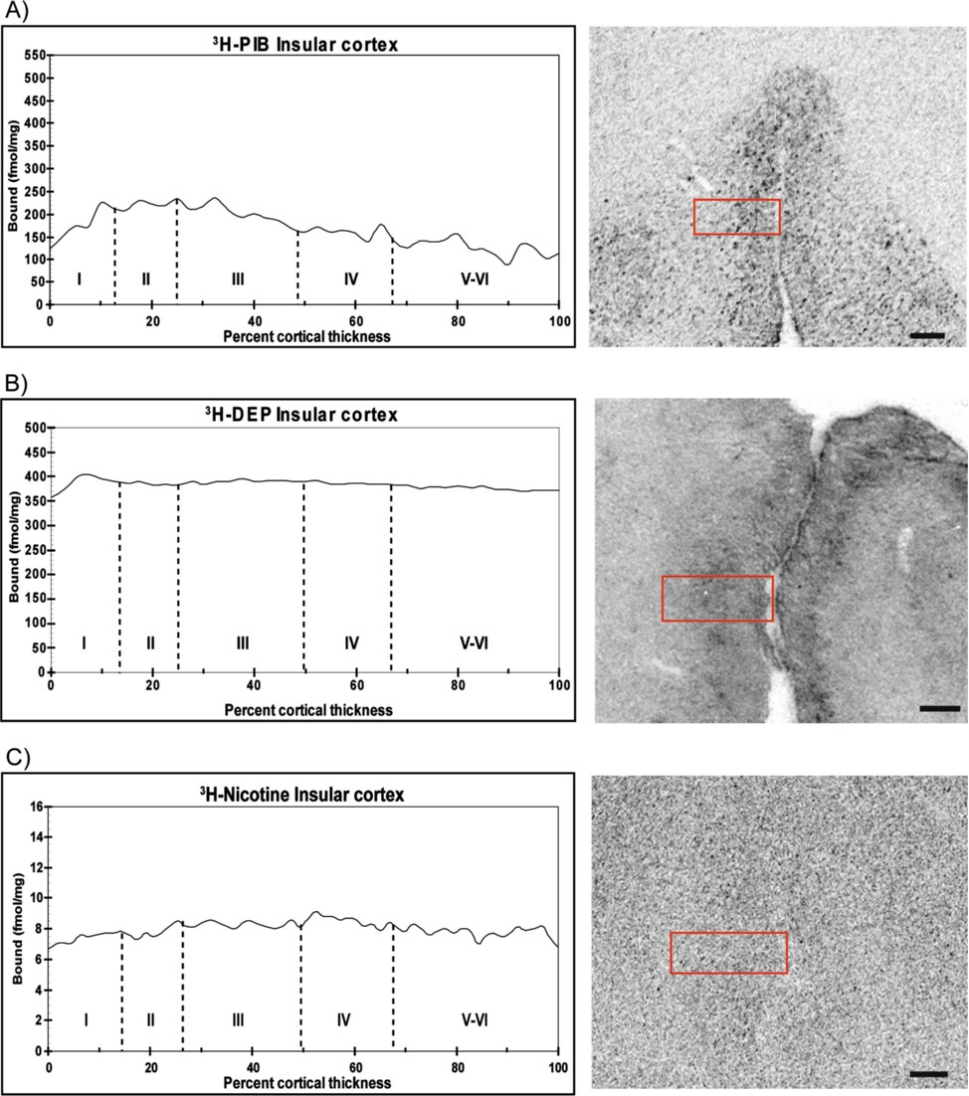
**Laminar binding distributions for (A) [**^**3**^**H]-PIB, (B) [**^**3**^**H]-l-deprenyl, and (C) [**^**3**^**H]-nicotine throughout the entire cortical depth of the insular cortex in an Alzheimer’s disease (AD) case.** The binding profiles are means created from three consecutive sections.

Next, we quantified the laminar distributions for each ligand in different layers of the cerebral cortex. [^3^H]-PIB showed high binding (350 to 500 fmol/mg tissue) in all layers of the superior frontal cortex (Figure 3A), while in contrast, [^3^H]-l-deprenyl binding showed high binding levels (400 fmol/mg tissue) only in the superficial layers (lamina I and II) of the frontal cortex (Figure 3B). The medial temporal gyrus and insular cortex exhibited similar high [^3^H]-l-deprenyl binding densities (400 fmol/mg tissue) in all layers, and equivalent lower [^3^H]-PIB binding densities (150 to 250 fmol/mg tissue) (Figures 4A,B and 5A,B). Among the cortical regions, the frontal cortex showed lower [^3^H]-nicotine binding (2 fmol/mg tissue), and more equal laminar binding in comparison to both the medial temporal gyrus and insular cortex (Figures 3C, 4C and 5C). The [^125^I]-α-bungarotoxin autoradiograms showed high background levels in cortical regions, which prevented further quantitative analysis of the specific binding (data not shown).

**Figure 6:**
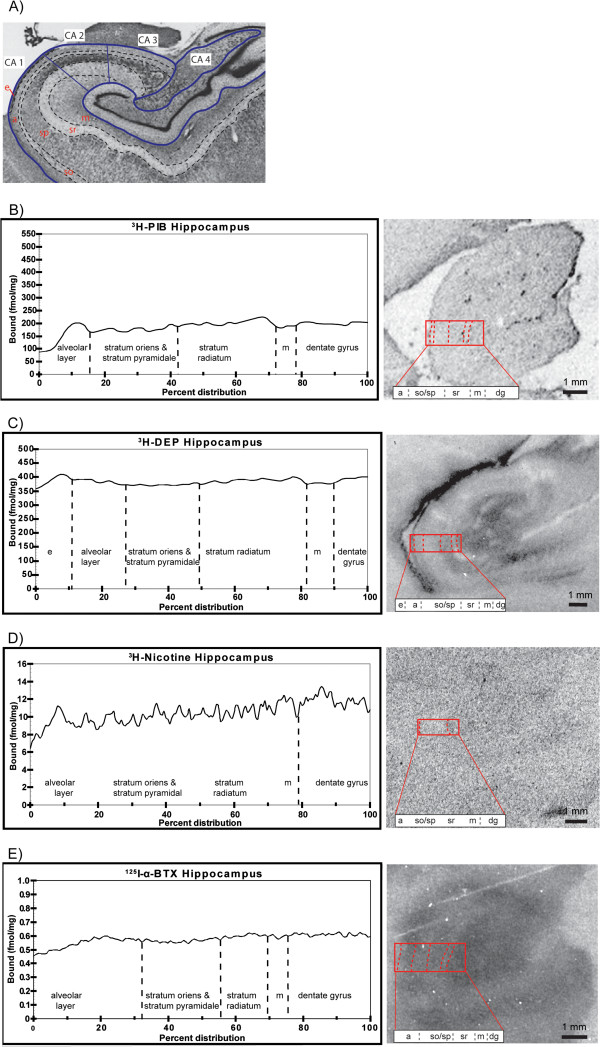
**Subregional binding distributions for [3H]-PIB, [3H]-L-deprenyl, and [3H]-nicotine and [125I]-α-bungarotoxin within the hippocampus of an Alzheimer’s disease (AD) case. (A)** An illustrative photomicrograph of a Nissl-stained section delineating the cytoarchitectonic layers of the hippocampus including the alveolar (*a*), stratum oriens (*so*), the stratum pyramidale (*sp*), stratum radiutum (*sr*), molecular layer of the dentate gyrus (*m*), and the *Cornu Ammonis* areas CA1 to CA4. Modified with permission from Teaktong *et al*. [65]. The binding distribution of **(B)** [^3^H]-PIB, **(C)** [^3^H]-l-deprenyl, **(D)** [^3^H]-nicotine, and **(E)** [^125^I]-α-bungarotoxin in the hippocampus of an Alzheimer’s disease (AD) case. The binding profiles are means created from three sections.

The hippocampal subregions examined are delineated in Figure 6A. [^3^H]-PIB showed low and uniform binding (180 to 200 fmol/mg tissue), while [^3^H]-l-deprenyl showed uniformly high binding (400 fmol/mg tissue) throughout the hippocampus (Figure 6B,C). [^125^I]-α-Bungarotoxin binding was observed mainly in the dentate gyrus, in contrast to [^3^H]-nicotine binding (Figure 6D,E).

### Topographical distributions of reactive astrocytes within the frontal cortex and hippocampus of AD brain by immunohistochemical staining

Neuropathological confirmation of the AD case showed characteristic AD neurodegenerative changes (deposition of Aβ and phosphorylated tau protein, the presence of activated microglia and reactive astrocytes) in the brain (Additional file 1: Figure S1).

**Figure 7:**
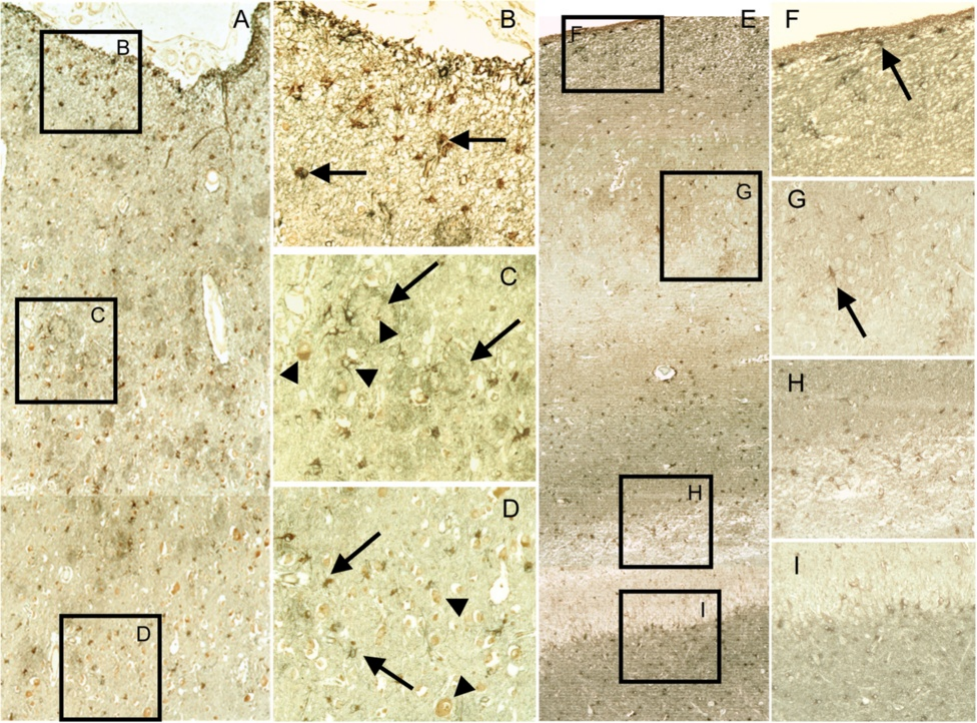
**The topographical distribution of reactive astrocytes within the superior frontal gyrus and hippocampus in an Alzheimer's disease case. (A)** Histological reconstruction of frontal gyrus gray matter made by the superimposition of identical areas of adjacent sections displaying reactive astrocytes with heterogenous morphologies in the different layers. 6F/3D β-amyloid (Aβ) plaques are in red color, reactive astrocytes positive for glial fibrillary acidic protein (GFAP+) are brown. Representative areas (B-D) are in the right column. **(B)** A uniform distribution of GFAP+ astrocytes (arrows) with small somata and densely stained neuropil in the superficial layers. **(C)** In the deeper layers, densely stained GFAP+ astrocytes and neuropil are concentrated around Aβ-neuritic plaques (arrows). **(D)** In the cortical lamina-VI bordering the white matter, GFAP+ astrocytes (arrows) were distributed more evenly as very few Aβ-neuritic plaques were observed in this region. **(E)** Histological reconstruction of the hippocampal CA1 and the dentate gyrus showing differences in the localization and intensity of immunoreactivity of GFAP+ astrocytes. GFAP+ astrocytes are brown, the α7nAChRs are gray. Representative areas are displayed in the corresponding inserts (F-I). **(F)** In the CA1 alveus and stratum oriens, intensely stained small somata of GFAP+/α7nAChRs astrocytes and networks were observed. **(G)** Throughout the stratum pyramidale, large somata of GFAP+ astrocytes without α7nAChRs (arrows) were found dispersed throughout GFAP+ network. **(H)** Differences in the intensity of immunoreactivity and the number of GFAP+ astrocytes were detected in the stratum lacunosum-moleculare (upper layer, strong staining) and the molecular layer of the dentate gyrus (bottom layer, weak staining). **(I)** In the granular layer, weakly stained GFAP+ astrocytes and network (top layer) were accompanied by very strong stained α7nAChR/GFAP+ astrocytes (bottom layer, gray). Videocapture was performed with ×10 (A, E) and ×20 (B-D, F-I) objective lenses magnification.

To relate the laminar [^3^H]-l-deprenyl autoradiography binding, the distribution of reactive astrocytes within different layers of the frontal cortex and hippocampal subregions in the single AD case was examined by immunohistochemistry with GFAP as a marker for astrocytosis. Differences in the localization and intensity of GFAP immunoreactive cells were found both within each region as well as between the regions (Figure 7). The distribution in the frontal cortex revealed a layer-specific localization of GFAP^+^ cells exhibiting heterogeneous morphologies (Figure 7A). A uniform distribution of reactive astrocytes with small somata and dense staining of GFAP^+^ neuropil was detected in the superficial (Figure 7B). In the deeper layers, GFAP^+^ astrocytic somata and densely stained neuropil were concentrated around Aβ plaques (Figure 7C). GFAP^+^ astrocytes were distributed more evenly in the cortical layer bordering the white matter (lamina VI), and very few Aβ plaques were observed in this region (Figure 7D).

A different staining pattern was observed in the hippocampus, where both GFAP^+^ astrocyte somata and processes surrounding 6 F/3D-positive Aβ plaques and GFAP^+^ astrocytes containing intracellular vesicles with 6 F/3D aggregates detected (Additional file 2: Figure S2).

In the CA1 alveus and stratum oriens of the hippocampus, we observed intensely stained small somata of GFAP^*+*^ astrocytes with immunoreactivity for α7 nAChRs as well as dense GFAP^*+*^/α7 nAChR-positive cellular networks (Figure 7E,F). Throughout the CA1 stratum pyramidal, large somata of GFAP^*+*^ astrocytes displaying no immunoreactivity for α7 nAChRs were found as well as dispersed GFAP^*+*^ network fibers (Figure 7G). Differences in the intensity of immunoreactivity and the number of GFAP^+^ astrocytes were detected in the CA1 stratum lacunosum-molecular and the molecular layer of the dentate gyrus (Figure 7H). In the granular layer, light brown stained somata of GFAP + astrocytes and weakly stained GFAP^+^ network fibers (top layer) were accompanied by very strong staining of α7 nAChR positive/GFAP^+^ astrocytes (gray hue, bottom layer) (Figure 7I).

## Discussion

Aβ deposition in the brain is a pathological hallmark of AD. In the present study *in vitro* binding studies in tissue homogenates showed significantly higher [^3^H]-PIB binding in the brain of a cohort of AD subjects compared to non-demented controls and thereby confirmed earlier observed high *in vivo*^11^C-PIB PET retention in cortex of AD patients compared to healthy controls [3]. Furthermore, in the current study *in vitro* imaging of the cortical laminar distribution pattern for fibrillar Aβ with [^3^H]-PIB autoradiography in a single AD case showed high binding in all layers of the frontal cortex. Earlier observations from quantitative immunohistochemical studies in AD autopsy brain have revealed high Aβ-plaque densities in layers II and III of the temporal and occipital cortices, while lower densities were reported in layers V and IV [10,28-31], but with larger plaque size in layer V [10], suggesting that Aβ plaque deposition may be intricately linked with cortical organization. Since ^3^H-PIB has been found to bind to multiple binding sites in AD frontal cortex; this could underlie the differences between ^3^H-PIB binding data and quantitative morphological measurement of Aβ plaques [10,32].

The detection of cerebral β-amyloidosis *in vivo* in patients with PET amyloid imaging tracers such as [^11^C]-PIB have been shown to correlate well with levels of fibrillar Aβ measured in AD brain at autopsy [6,33]. Measurement of the distribution of [^11^C]-PIB retention based on cytoarchitectonic subtypes of the cerebral cortex demonstrated that the neocortex with more fully laminar differentiation showed abundant [^11^C]-PIB retention, while phylogenetically older limbic areas, such as the allocortex and the periallocortex, with fewer laminae appeared less vulnerable to [^11^C]-PIB retention, when comparing AD patients with healthy control subjects [34]. Non-random Aβ plaque distribution within cortical areas could imply that specific neurons or spatial arrangements of neuronal networks can serve as a substrate for plaque aggregation, where factors such as the regional concentration of Aβ, in turn determines the amount of plaque load in a given area, while the local architectonics of the cortex determines the distribution pattern among brain regions. This differential cytoarchitectonic vulnerability to Aβ deposition may underlie the progressive neuropathological alterations involved in the hierarchical organized central nervous system in the pathogenesis of AD.

Different forms of Aβ in the brain can elicit activation and recruitment of microglia and astrocytes. These cells play a central role in the cellular response to pathological lesions and exercise both neuroprotective and neurotoxic functions in the brain, mediated by the secretion of cytokines and chemokines and the binding of these to their specific receptors [34-37]. Here, we demonstrated increased levels of both activated microglia and reactive astrocytes in AD frontal cortex and hippocampus as measured by [^3^H]-PK11195 and [^3^H]-l-deprenyl binding, respectively. *In vivo* PET studies with [^11^C]-PK11195 have demonstrated both increased and unchanged binding in AD patients compare to healthy subjects [38,39] and there might be different explanations for discrepancies in findings such as different used PET protocols, variation in sensitivity of the PET tracer as well as differences in patient material. Deprenyl is a selective irreversible monoamine oxidase B (MAO-B) inhibitor and is considered to be a sensitive marker for measuring astrocytosis in the brain, since the MAO-B enzyme is upregulated in reactive astrocytes, giving rise to increased regional uptake of deprenyl [40]. A strong correlation between [^3^H]-l-deprenyl binding and MAO-B activity in both AD and non-demented control autopsy brain tissue has previously been reported [41,42]. Interestingly enough *in vivo* PET studies have shown higher ^11^C-deuterium-l-deprenyl binding in brain of patients with mild cognitive impairment (MCI) than AD and controls [17]. The observation might indicate a difference in astrocytes’ properties in early and later stages of disease. In the present study, we observed a regional difference between [^3^H]-l-deprenyl and [^3^H]-PK11195 in increase in binding sites in AD brains in comparison to control brains. Interestingly, [^3^H]-l-deprenyl laminar binding in the single AD case was found to be relatively higher in the superficial cortical layers of the frontal cortex in comparison to binding in the deeper cortical layers. Immunohistochemical staining in the same AD case confirmed abundant GFAP^+^ reactive astrocytes surrounding Aβ plaques in the superficial layers of the frontal cortex. These findings are in agreement with earlier reports on the pattern of gliosis in AD in layers II to III and V [15].

It has been claimed that neurofibrillary tangles and Aβ plaques should favor the same cortical layers as astroglia [10,30,31,43]. The laminar pattern of [^3^H]-PIB binding in cortex may thus suggest binding to additional forms of Aβ than plaques. The high binding of both [^3^H]-PIB and [^3^H]-deprenyl in the frontal cortex suggests that there may be a close regional association between elevated astroglia and fibrillar Aβ deposition, which is consistent with our earlier observations in which a positive correlation between regional brain [^11^C]-PIB retention, [^3^H]-PIB binding, and the total number of GFAP^+^ immunoreactive astrocytes was found in the same patient [6].

The hippocampus, however, revealed a different laminar pattern, where high [^3^H]-l-deprenyl, but low [^3^H]-PIB binding densities were observed in all hippocampal subregions. The observation is in agreement with the low amyloid but high gliosis in the hippocampus as described by Beach and colleagues [15]. The hypothesis of Rogers and Morrison [10] predict a cascade of pathology wherever there is a cascade of convergent cortical input, and this is precisely the case Hyman *et al*. [44], showed with their findings of cell-specific pathology isolates the hippocampal formation in AD cases. Soluble Aβ oligomers have received much attention and it is argued that these assemblies play a major role in mediating neuronal damage in comparison to their insoluble counterparts [1,45]. It is not known whether the concentration of Aβ oligomers or intracellular Aβ is significantly greater in the hippocampus compared to cortical regions since studies to date characterizing various Aβ oligomer assemblies in AD postmortem brain, have mostly been performed using cortical brain extracts [46-48]. Non-fibrillar Aβ aggregates are not readily detected *in vivo* with the currently available amyloid PET tracers, and it is possible that these soluble assemblies could underlie the increased astrogliosis detected within the hippocampus in the current study.

Astrocytes can take up Aβ in complex with apolipoprotein E (ApoE) as well as degrade Aβ by neprilysin [49-51]. Different types of astroglial cells can be distinguished in AD brain, including reactive, hypertrophic astrocytes in the vicinity of Aβ plaques [10] and neurofibrillary tangles (NFTs) [52], Aβ-containing astrocytes that may possibly be involved in the removal of diffuse plaques and fleecy amyloid [29,53,54], and functionally impaired astrocytes with deficits in gene or protein expression [49]. Atrophy of astrocytes occurring in the early stage of AD is suggested to influence synaptic function and cognition [55].

Neuropathological changes in the brain during the course of AD may disrupt the columnar organization of the cerebral cortex, which in turn result in changes in interlaminar astrocytic processes and modulation of their function [15,56]. A decline in regional cerebral glucose metabolism, determined by [^18^F]-fluorodeoxyglucose (FDG) PET, in an AD patient 16 months before death correlated with cortical neuronal loss and with intense staining of GFAP^+^ cells in cortical areas at autopsy [57].

In addition to their role in neuroinflammatory processes, astrocytes in the central nervous system also provide structural and trophic support and are actively involved in the regulation of neuronal and synaptic activity with the purpose of maintaining overall brain homeostasis [58,59]. Astrocytes that undergo both morphological and structural changes during AD neurodegeneration neglect their neurosupportive functions as pathogenesis advances, rendering neurons vulnerable to excitotoxicity and oxidative stress [49,50,60,61]. Therefore, it is likely that a strong inflammatory response triggered by the neurodegenerative changes in the AD brain, accelerate and drive the degenerative pathology, contributing to disease progression and chronicity.

An increase in regional [^3^H]-l-deprenyl binding was recently reported in autopsy cases with lower Braak stages compared to cases with higher Braak stages [62]. However, astrocytosis is a complex process and poorly understood. Whether the reactive astrocytes in AD brain can adopt different states of activation and whether increased MAO-B activation *in vivo* or [^3^H]-l-deprenyl binding *in vitro* reflects a certain type of astrocytes warrants future study.

In a recent study using rodent models of ischemic stroke and neuroinflammation, Barres and colleagues performed gene expression profiling analysis of populations of reactive astrocytes isolated at various time points after induction, and demonstrated that reactive astrocyte phenotype strongly depended on the type of inducing injury [63].

The different laminar distribution patterns for Aβ and astrocytosis in AD brain regions demonstrated in the present study are indicative of two parallel processes, which may follow a different time course, and show a regional variability that depends on the initiating insult triggered in specific areas of the brain. Although the postmortem imaging findings reported here were in line with the homogenate binding studies, a weakness of the current study is that the autoradiographical investigation was carried out in solely a single AD case subject. However, this kind of technique examining laminar distributions in detail is a lengthy and time consuming process, and does not allow simultaneous processing of many cases. Our present findings in hemisphere brain have also been validated in small tissue sections and gave similar findings although the resolutions in the smaller sections were lower (data not shown).

A growing number of studies have pointed to an involvement of brain nAChRs and neuroinflammatory processes in Aβ pathology. Both neurons and glia cells express several nAChRs including the two major subtypes in the brain, namely α4β2 and α7 nAChRs [64]. Earlier, we demonstrated that a reduction of α4β2 nAChRs in AD cortex is associated with high levels of fibrillar Aβ as well as higher molecular weight oligomeric Aβ assemblies [6,46]. Interestingly, we observed in the present study that increased distribution of fibrillar Aβ in the frontal cortex was associated with lower laminar [^3^H]-nicotine binding compared to other cortical regions, suggesting that Aβ may induce a selective vulnerability of some areas of cortical projections in the brain involving specific or discrete neural systems.

While there is a reduced density of α7 nAChRs on neuronal cells, an increased number of these receptors have been found on astrocytes as measured in AD autopsy brains [27,65,66]. It is known that astrocytes modulate neuronal activity partly via ion channels and through the neurotransmitter receptors they express [67]. The α7 nAChRs expressed on astrocytes could, thus, influence the excitability of astrocytes and their ability to propagate Ca^2+^-mediated signaling mechanisms. The amount of soluble and/or non-soluble Aβ forms as well as their internal versus external localization [68] in different brain regions could also influence intermediate processes between α7 nAChRs, glia and neurons, as implicated by recent findings from our group [6,69].

## Conclusions

In summary, we demonstrate an elevation in astrogliosis both in areas with high and low fibrillar Aβ burden and greater atrophy, which may suggest that different types of reactive astrocytes are associated with the pathophysiological processes in the AD brain. It is important to continue to study these processes *in vivo*, in order to obtain a better understanding of how astrocytes interact with neuronal network function, and to resolve whether these cells contribute to cognitive decline in AD.

## Competing interests

The authors declare that they have no competing interests or potential conflict of interest related to this study.

## Authors’ contributions

The work presented here was carried out in collaboration between all authors. AM, P-GG and AB carried out most of the laboratory experiments. LV and WY performed the immunohistochemical analysis and IN the neuropathological investigation. All authors contributed to data analysis of the data and interpretation of the results. AM, P-GG and AN conceived the idea for the study, and helped in designing methods and experiments. AN critically supervised the complete study. All the authors read and approved the final revised manuscript.

## Supplementary Material

Additional file 1: Figure S1 Neuropathology in the brain obtained from an Alzheimer’s disease case used for autoradiography studies showing immunoreactivity in the frontal cortex and hippocampus, respectively, for: (A-B) β-amyloid (Aβ), 6 F/3D; (C-D) hyperphosphorylated tau protein, AT8; (E-F) activated microglia, CD68; and (G-H) reactive astrocytes, glial fibrillary acidic protein (GFAP). Click here for file

Additional file 2: Figure S2 Immunohistochemical staining of GFAP^+^ reactive astrocytes and Aβ aggregates in the hippocampus of the Alzheimer’s disease case. In the hippocampus, both GFAP + astrocyte somata and processes (brown) surrounding 6 F/3D-positive Aβ plaques *(red)* (A) and glial fibrillary acidic protein-positive (GFAP^+^) astrocytes containing intracellular vesicles with 6 F/3D Aβ (B) were detected. GFAP^+^ cells are indicated by arrows. Click here for file
