# Supplementary figures and images for: 3H-Deprenyl and 3H-PIB autoradiography show different laminar distributions of astroglia and fibrillar β-amyloid in Alzheimer brain
Source: J Neuroinflammation. 2013 Jul 23;10:90. doi: 10.1186/1742-2094-10-90 (PMC3733895; doi:10.1186/1742-2094-10-90)

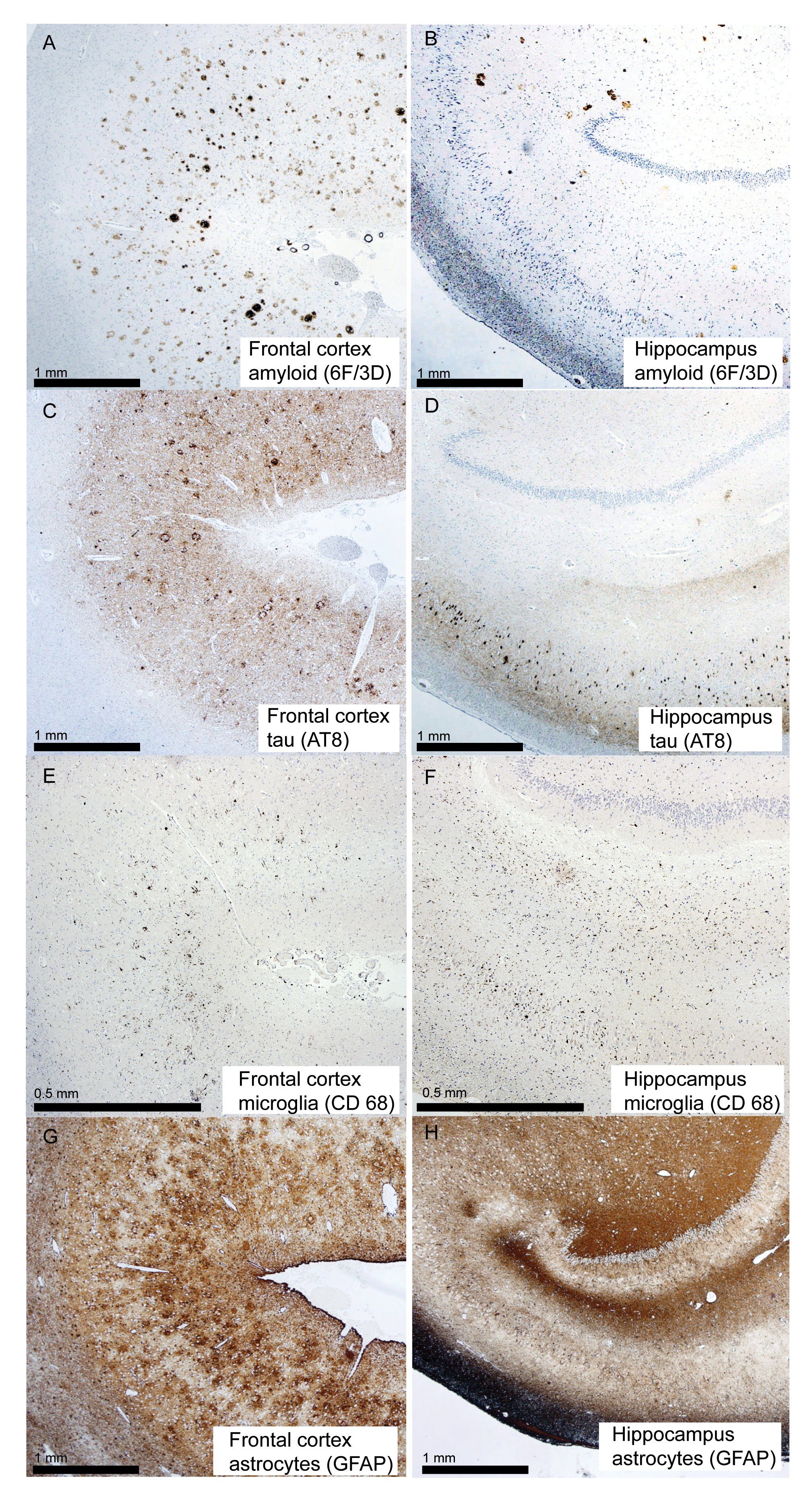

Supplement: Additional file 1: Figure S1 — Neuropathology in the brain obtained from an Alzheimer’s disease case used for autoradiography studies showing immunoreactivity in the frontal cortex and hippocampus, respectively, for: (A-B) β-amyloid (Aβ), 6 F/3D; (C-D) hyperphosphorylated tau protein, AT8; (E-F) activated microglia, CD68; and (G-H) reactive astrocytes, glial fibrillary acidic protein (GFAP). [file 1742-2094-10-90-S1.zip › Supplementary Fig 1 .tif]

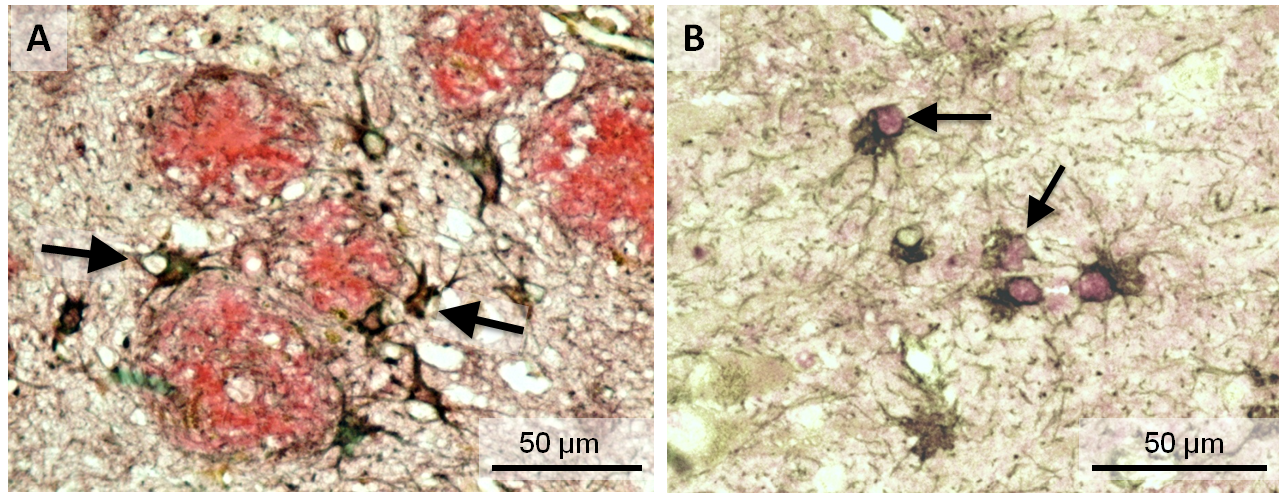

Supplement: Additional file 2: Figure S2 — Immunohistochemical staining of GFAP+ reactive astrocytes and Aβ aggregates in the hippocampus of the Alzheimer’s disease case. In the hippocampus, both GFAP + astrocyte somata and processes (brown) surrounding 6 F/3D-positive Aβ plaques (red) (A) and glial fibrillary acidic protein-positive (GFAP+) astrocytes containing intracellular vesicles with 6 F/3D Aβ (B) were detected. GFAP+ cells are indicated by arrows. [file 1742-2094-10-90-S2.tiff]
